# *Lingguizhugan* Decoction Protects against High-Fat-Diet-Induced Nonalcoholic Fatty Liver Disease by Alleviating Oxidative Stress and Activating Cholesterol Secretion

**DOI:** 10.1155/2017/2790864

**Published:** 2017-12-31

**Authors:** Lili Yang, Weili Lin, Colleen A. Nugent, Shijun Hao, Haiyan Song, Tao Liu, Peiyong Zheng

**Affiliations:** ^1^Institute of Digestive Diseases, Longhua Hospital, China-Canada Center of Research for Digestive Diseases (ccCRDD), Shanghai University of Traditional Chinese Medicine, Shanghai 200032, China; ^2^Key Laboratory of Computational Biology, CAS-MPG Partner Institute for Computational Biology, Shanghai Institutes for Biological Sciences, Chinese Academy of Sciences, Shanghai 200032, China; ^3^University of Chinese Academy of Sciences, Beijing 100049, China; ^4^Digestive Diseases and Nutrition Center, Women and Children's Hospital of Buffalo, Department of Pediatrics, The State University of New York at Buffalo, Buffalo, NY 14214, USA

## Abstract

**Background:**

Nonalcoholic fatty liver disease (NAFLD) has become a leading cause of liver transplantation. *Lingguizhugan* decoction (LGZG), a classical Chinese herbal formula, has beneficial effects on NAFLD animal models. Our study examined the impact of LGZG on hepatic global transcriptome of high-fat-diet-induced NAFLD rats.

**Methods:**

Three groups of Wistar rats were included: normal, NAFLD model, and LGZG-treated NAFLD groups. Four weeks for the treatment, liver tissues were harvested for RNA sequencing. Differentially expressed genes (DEGs) and enriched pathways were detected on hepatic global transcriptome profile. Real-time PCR validated the regulatory patterns of LGZG on NAFLD rats.

**Results:**

DEGs between the NAFLD model and normal groups indicated the elevated peroxisome proliferator-activated receptor (PPAR) and hedgehog signaling pathways in NAFLD rats. In bile secretion pathway, genes involved in cholesterol secretion were activated by LGZG treatment. Increased expression of antioxidant *OSIGN1* and decreased expression of genes (*AHR*, *IRF2BP2*, and *RASGEF1B*) that induce oxidative stress and inflammation were observed in NAFLD rats treated with LGZG. The regulatory patterns of LGZG treatment on these oxidative stress-related genes were confirmed by real-time PCR.

**Conclusion:**

Our study revealed a “two-hits-targeting” mechanism of LGZG in the treatment for NAFLD: alleviating oxidative stress and activating cholesterol secretion.

## 1. Introduction

Nonalcoholic fatty liver disease (NAFLD) encompasses a spectrum of pathological conditions, including simple steatosis, nonalcoholic steatohepatitis (NASH), fibrosis, and cirrhosis. NAFLD has been increasingly prevalent worldwide and has become a leading cause of liver transplantation, along with increasing obesity rate and metabolic syndrome [1]. According to the “two-hits” hypothesis of NAFLD pathogenesis, the first hit is represented by lipid accumulation in the hepatocytes, after which oxidative stress leads to severe NASH [2]. Additionally, recent studies demonstrated that other risk factors also contribute to the development of severe NASH, including altered gut microbiota [3, 4], endogenous alcohol metabolism [5–7], and endoplasmic reticulum stress [8]. Currently, many potential targets for the treatment of NAFLD are identified, including lipid metabolism, oxidative stress, inflammation, fibrosis, and altered gut microbiota [9]. However, clinical trials exclusively aimed at only one of these targets at a time and achieved limited effects. For example, vitamin E therapy targets the “second hit”—oxidative stress, and pioglitazone therapy targets insulin resistance. Both of these two therapies decrease serum AST/ALT and reduce lobular inflammation. They, however, have no impact on insulin resistance, portal inflammation, or liver fibrosis and did not obtain satisfactory sustained results [10].

Given NAFLD is a consequence of multiple risk factors, especially the major “two hits”—lipid accumulation and oxidative stress. A therapeutic strategy that targets both of “two hits” or several hits simultaneously could be more effective than one single therapeutic target. Traditional Chinese medicine (TCM) has been increasingly applied as the potential anti-NAFLD drugs and exhibited multipronged preventive and therapeutic effects [9, 11, 12]. LGZG is a classic TCM formula, which is a mixture of four herbs: *Poria*, *Ramulus Cinnamomi*, *Atractylodes macrocephala Koidz*, and *Radix Glycyrrhizae*. LGZG has a long time of clinical practice in the treatment of chronic congestive heart failure. In recent years, LGZG has been effectively used to treat obesity and hyperlipidemia [13] and our previous study showed that LGZG has a benefit in treating NAFLD [14].

TCM is an ancient medical practice system which emphasizes the integrity of the entire human body, and it usually exerts therapeutic effects via multiple targets or pathways. Recently, high-throughput omics technologies, especially transcriptomics RNA sequencing, have been increasingly applied in TCM research and revealed multipronged therapeutic mechanisms of TCM [11, 15]. Herein, to better understand the therapeutic mechanisms of LGZG, we examined the effects of LGZG treatment on the hepatic global transcriptome of HFD-induced NAFLD rats. Combined with real-time PCR validation, we identified a “two-hits-targeting” mechanism for LGZG in the treatment of NAFLD.

## 2. Materials and Methods

### 2.1. Experimental Animals and Treatment

This study was reviewed and approved by the Animal Experiment Ethics Committee of Shanghai University of TCM and carried out in accordance with their recommendations. A total of 24 male Wistar rats with weights of 130 g ± 10 g in specific pathogen free (SPF) grade were purchased from Shanghai Si-Lai-Ke Experimental Animal Ltd. (Shanghai, China). Animals were randomly divided into three groups (eight rats per group): normal, NAFLD model, and LGZG-treated NAFLD groups. Rats in the normal group were fed with a standard diet. Rats in the NAFLD model and LGZG-treated NAFLD groups were fed with HFD, which consists of 10% lard oil, 2% cholesterol, and 88% standard chow. Rats in the LGZG-treated NAFLD group received a dosage of 10 mL/kg/d (pure solution) via drinking freely. The dosage was 6 g crude medical material per kilogram body weight, approximately seven times of the standard dosage in practice, on the basis of the dose-equivalence equation between rats and humans [16]. Treatment lasted for four weeks. After a 12-hour fast, the animals were sacrificed under pentobarbital sodium (2%, 5.5 mL/kg) anesthesia. Liver tissues were harvested for subsequent analysis.

### 2.2. Drug Preparation

LGZG decoction is comprised of four Chinese herbs: *Poria* (20 g), *Ramulus Cinnamomi* (15 g), *Rhizoma Atractylodis Macrocephalae* (15 g), and *Radix Glycyrrhizae* (10 g). The dosage is determined according to the test book of “*The Hndouts of JinguiYaoyue*.” All herbs were purchased from Longhua Hospital affiliated to Shanghai University of TCM. LGZG decoction was made according to conventional TCM decocting methods [14]. Briefly, all herbs were boiled with 500 mL water after 30 min of soaking. After 20 min, the liquid was transferred by filtration as a first dose of medicine. The remaining of filtration was boiled after adding 400 mL water, and then liquid was transferred by filtration to make a second dose. Two doses were mixed to form 100 mL (pure solution) final decoction. The quality of LGZG was controlled with high-performance liquid chromatography (HPLC). HPLC-grade reagents were purchased from Burdick & Jackson. An Agilent 1100 HPLC system consisting of a G1354A pump, a G1313A autosampler, and a UV/VIS Photodiode Array G1315B detector was used for all analyses (Figure S1).

### 2.3. RNA Sequencing Analysis

Liver tissue from the right lobe was collected after four weeks of LGZG treatment as described above. Total RNA was isolated with NanoPhotometer spectrophotometer (IMPLEN, CA, USA) and qualified on the Qubit RNA Assay (Qubit 2.0 Fluorometer, Life Technologies, CA, USA). The RNA libraries were sequenced on IlluminaHiseq 4000 platform with paired-end 150 base pair long reads. Clean data were obtained from raw data by removing reads containing adapter, N base, and low-quality reads with NGS QC Toolkit (version: 2.3.3). Clean data were mapped to the reference genome of R.norvegicus6.0 and estimated for gene expression level using TopHat2 and cufflinks (version: 2.2.1).

### 2.4. Real-Time PCR

*OSGIN1*, *IRF2BP2*, *AHR*, and *RASGEF1B* mRNA levels were determined by real-time PCR. Primers were designed with the primer premier 5.0 software (Table S1). Total RNA of liver tissues was extracted with TRIzol reagent (Invitrogen, USA). The concentration of RNA was measured with NanoDrop 2000 (Thermo Scientific, USA). Quantitative measurement was performed with the Premix Ex Taq kit (TakaRa) according to the manufacturer's instructions on Applied Biosystems StepOne Plus Sequence Detection System. The real-time cycler conditions were as follows: first denatured at 95°C for 30 s and then amplified with 40 cycles (each cycle was denaturated at 90°C for 5 s and annealing/extension at 60°C for 30 min). Product purity was determined by dissociation curve analysis. Gene expression was quantified relative to the values of the control group after adjusting for *β*-actin by the 2^−ΔΔCT^ method [17].

### 2.5. Statistical Analysis

Differentially expressed genes (DEGs) were identified by cuffdiff (version: 2.2.1), with a *Q* value < 0.05 and an absolute value of log2 fold change > 0.58. Pathway enrichment analysis was performed with clusterProfiler R package (version: 2.4.3) with *P* value < 0.05. Data were denoted as mean ± standard deviation (SD). In biochemical analysis, statistical analysis was performed with one-way analysis of variance (ANOVA) and Dunnett's test. In RT-PCR validation, statistical analysis was performed with Mann–Whitney test with a two-tailed distribution. *P* values < 0.05 were considered statistically significant. Statistical analysis was performed in R 3.2.3 software.

## 3. Results

### 3.1. Genes and Pathways Associated with NAFLD Pathology

**Figure 1:**
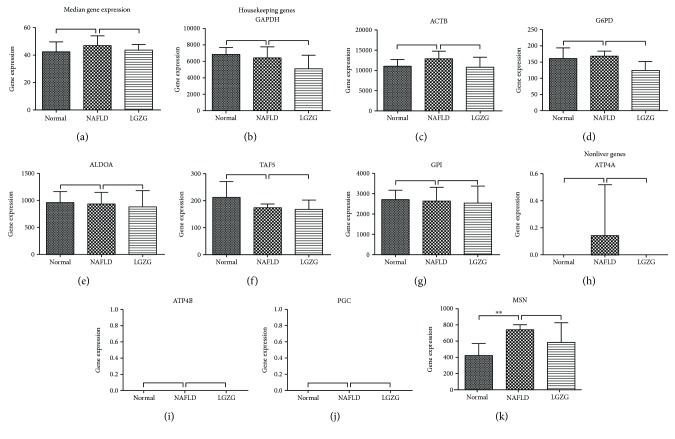
Median gene expression (a) was similar among all study groups. Housekeeping genes *GAPDH* (b), *ACTB* (c), *G6PD* (d), *ALDOA* (e), *TAF* (f), and *GPI* (g) and nonliver genes *ATP4A* (h), *ATP4B* (i), *PGC* (j), and *MSN* (k) exhibited similar expression among all study groups. ^∗∗^*Q* value < 0.01 calculated from cuffdiff.

**Figure 2:**
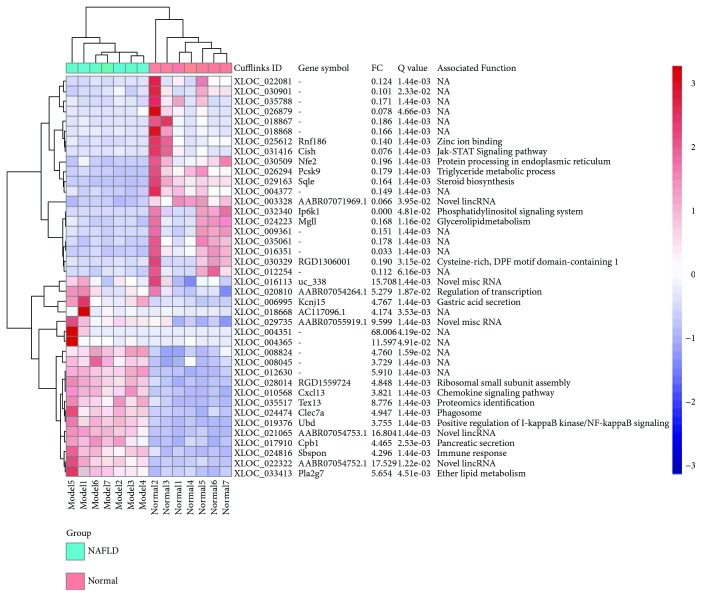
Differential expression of top 40 NAFLD-regulated genes. The samples are broadly divided into two groups, normal and NAFLD model groups. The color scale shown at the top right illustrates the relative expression level of the genes across all samples. FC, fold change between NAFLD model and normal groups.

Expression levels of about 22,077 genes of rat livers were quantified on a global RNA sequencing. Similar median gene expression and expression levels for housekeeping genes and nonliver genes are indicative of the qualified RNA-seq data in the present study (Figure 1). A total of 931 genes were differentially expressed between the NAFLD model and normal groups, including 494 upregulated and 437 downregulated genes. These 931 DEGs were regarded as NAFLD-regulated genes. The top 40 regulated genes (20 most upregulated and 20 most downregulated) were depicted in Figure 2. These genes are known to be associated with lipid metabolism, including triglyceride metabolic process (*PCSK9*), steroid biosynthesis (*SQLE*), glycerolipid metabolism (*MGLL*), ether lipid metabolism (*PLA2G7*), and phosphatidylinositol signaling system (*IP6K1*). Additionally, the chemokine *CXCL13*, ubiquitin *UBD*, and somatomedin *SBSPON* play roles in inflammatory responses, and their expressions were elevated in NAFLD rats (Figure 2).

**Figure 3:**
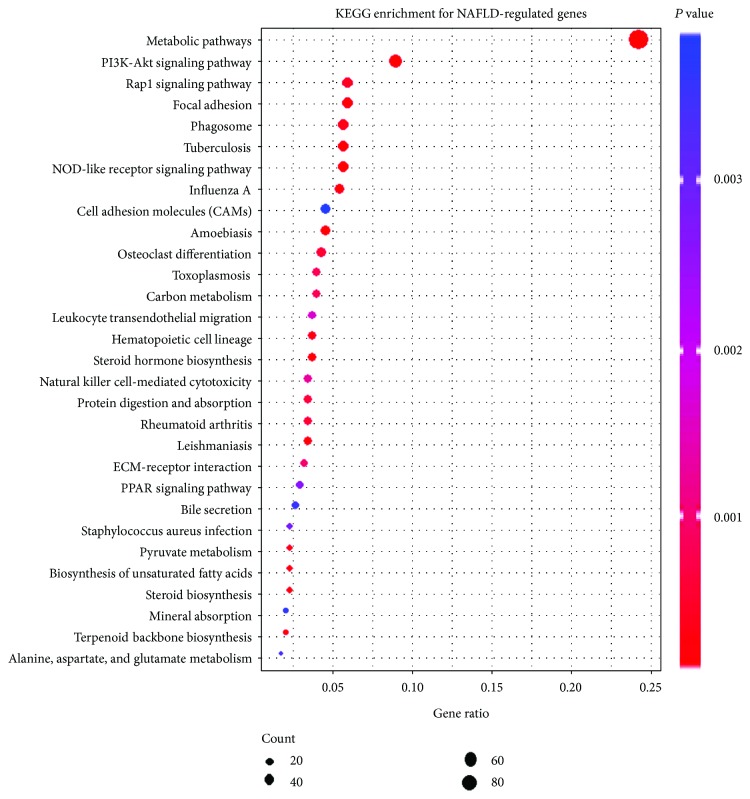
Scatter plot of the top 30 enriched KEGG pathways for NAFLD-regulated genes. The *x*-axis represents the ratio of NAFLD-regulated gene and all gene numbers annotated in this KEGG pathway. The *y*-axis is enriched KEGG pathways. *P* value was calculated from hypergeometric test. A smaller *P* value indicates higher significance (*P* < 0.05).

**Figure 4:**
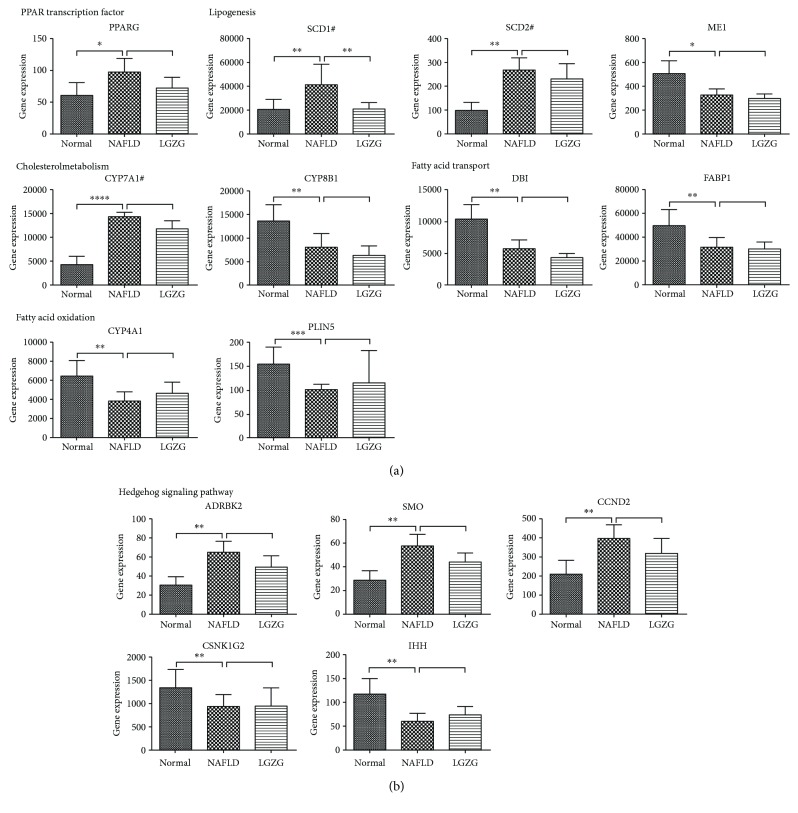
Differential gene expression of (a) PPAR and (b) hedgehog signaling pathways in a NAFLD rat model. ^∗^*Q* value < 0.05; ^∗∗^*Q* value < 0.01; ^∗∗∗^*Q* value < 0.001; ^∗∗∗∗^*Q* value < 0.0001 calculated from cuffdiff. ^#^Rate-limiting enzyme.

Further, 76 KEGG pathways were enriched with NAFLD-regulated genes (Table S2). The top 30 enriched KEGG pathways were shown in Figure 3. In addition to several well-known pathways that are related to NAFLD pathology (including fatty acid elongation, AMPK signaling pathway, and NF-kappa B signaling pathway), peroxisome proliferator-activated receptor (PPAR) and hedgehog (Hh) signaling pathways (Table S2) are of special interest because of their essential roles in hepatic fibrosis of NAFLD progression. As to individual genes of PPAR signaling pathway (Figure 4(a)), NAFLD rats exhibited increased expression of one of the nuclear receptors—*PPARG*, which regulates lipogenesis and cholesterol metabolism and represents a good candidate gene for NAFLD [18]. Consistently, increased expression of stearoyl-CoA desaturase (*SCD1* and *SCD2*) was observed in NAFLD rat livers, which are regulated by *PPARG* and the rate-limiting enzymes in lipid biosynthesis [19, 20]. *CYP7A1*, a rate-limiting enzyme in cholesterol metabolism, was upregulated in NAFLD rats. However, some target genes of *PPARG* were downregulated in NAFLD rats. And these decreased expressions of the target genes resulted in impaired fatty acid transport (*DBI* and *FABP1*) and oxidation (*CYP4A1*), which contributed to fatty acid accumulation in the NAFLD liver.

G protein-coupled transmembrane receptor Smoothened (*SMO*) is one of important components of canonical Hh signaling pathway. Our results showed upregulation of *SMO* in NAFLD rats, compared with normal rats. A trend of decreased expression of *SMO* was shown in LGZG-treated rats. In addition, *ADRBK2* (upstream regulator of *SMO*) and *CCND2* (Hh downstream target gene) were also significantly elevated in NAFLD rats (Figure 4(b)).

### 3.2. Genes and Pathways Mediating the Therapeutic Effects of LGZG

**Figure 5:**
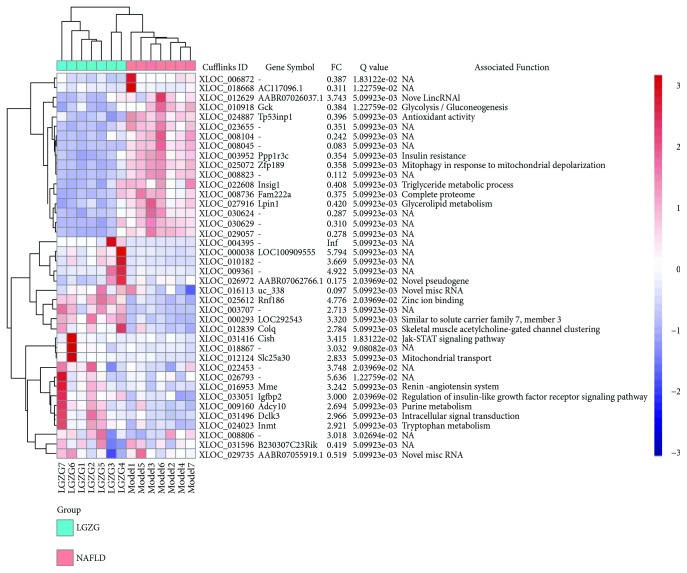
Differential expression of top 40 LGZG-regulated genes. The samples of the NAFLD model group formed a clade and were separated from the samples of the LGZG-treated NAFLD groups. The color scale shown at the top right illustrates the relative expression level of the genes across all samples. FC, fold change between LGZG-treated NAFLD and NAFLD model groups.

**Figure 6:**
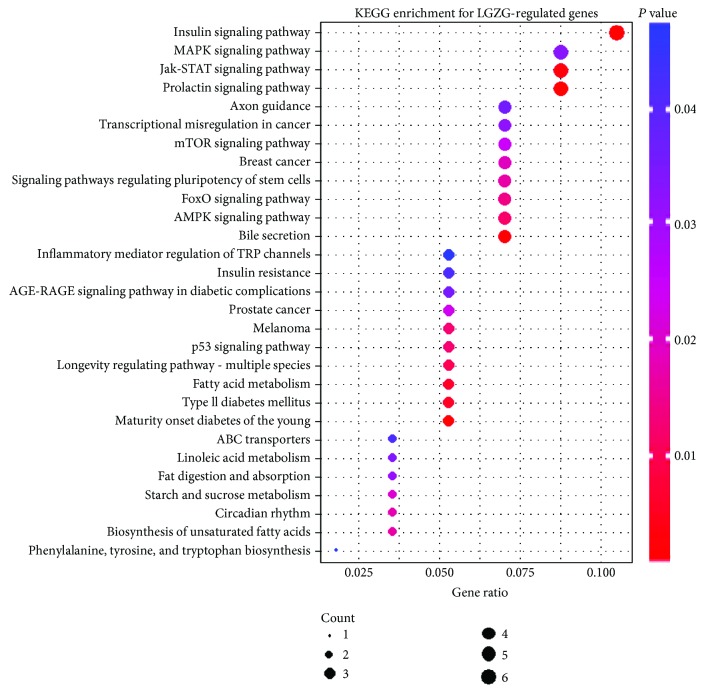
Scatter plot of the enriched KEGG pathways for LGZG-regulated genes. The *x*-axis represents the ratio of LGZG-regulated gene numbers annotated in this KEGG pathway to all gene numbers annotated in this KEGG pathway. The *y*-axis is enriched KEGG pathways. *P* value was calculated from hypergeometric test. A smaller *P* value indicates higher significance (*P* < 0.05).

Compared with the NAFLD model group, elevated expression of 110 genes and decreased expression of 89 genes were observed in the livers of the LGZG-treated NAFLD rats. These total 199 genes were considered as LGZG-regulated genes.  Figure 5 showed the top 40 LGZG-regulated genes (20 most upregulated and 20 most downregulated). The LGZG-treated NAFLD rats exhibited reduced expression of *INSIG1* and *LPIN1*, which indicated the decreased cholesterol biosynthesis and triglyceride accumulation in the liver. Pathway enrichment analysis was performed with LGZG-regulated genes and unearthed 29 enriched pathways (Figure 6, Table S3). In bile secretion pathway, genes required for cholesterol secretion were elevated, including *ABCG8*, *ABCG5*, and *NCEH1*. Some metabolism and signaling pathways were also enriched, including fatty acid metabolism, Jak-STAT, and FoxO signaling pathways.

**Figure 7:**
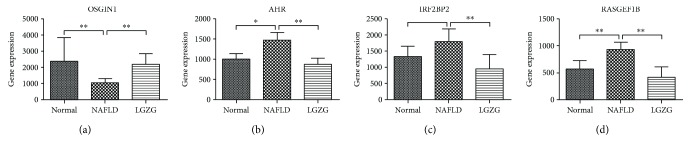
Differential gene expression of LGZG-regulated genes. ^∗^*Q* value < 0.05; ^∗∗^*Q* value < 0.01 calculated from cuffdiff.

Furthermore, four (*OSGIN1*, *AHR*, *IRF2BP2*, and *RASGEF1B*) were shown to be strongly regulated upon LGZG therapy, which may play potential roles in NAFLD physiopathology (Figure 7, Table 1). These four genes also showed differential expression changes in NAFLD rats, compared with normal rats. Also, the abnormal expression change in NAFLD rats was significantly reversed by the LGZG therapy. Thus, they were considered as the important target genes of LGZG.

### 3.3. Validation of LGZG-Regulated Genes with Real-Time PCR

**Figure 8:**
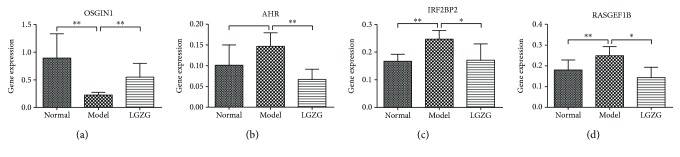
Differential gene expression of LGZG-regulated genes was examined by real-time PCR. Plotted values are the mean ± SD of mRNA expression levels in the livers of normal rats (normal), NAFLD model rats, and NAFLD rats treated with LGZG decoction (LGZG). *N* = 6 per group. ^∗^*P* < 0.05; ^∗∗^*P* < 0.01 Mann–Whitney test.

Expression levels of four LGZG-regulated genes were validated with real-time PCR, including *OSGIN1*, *AHR, IRF2BP2*, and *RASGEF1B* (Figure 8). The real-time PCR results confirmed that the expression of *OSGIN1* was reversely elevated, and the expression levels of *IRF2BP2*, *AHR*, and *RASGEF1B* were inversely suppressed under the LGZG treatment.

## 4. Discussion

**Figure 9:**
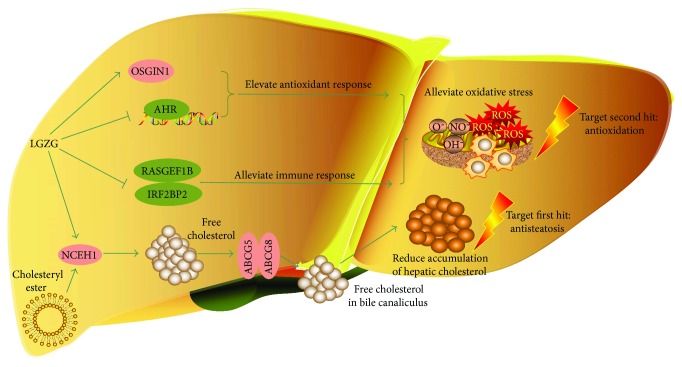
Summary of the “two-hits-targeting” therapeutic mechanisms of LGZG for NAFLD. Data presented in this study supports two independent mechanisms: (i) LGZG activates antioxidant (*OSGIN1*) and suppresses prooxidant and proinflammatory genes (*AHR*, *IRF2BP2*, and *RASGEF1B*), causing alleviated oxidative stress, and (ii) LGZG induces cholesterol secretion genes in the liver, leading to reduced accumulation of liver lipid. These mechanisms reflect that LGZG treatment prominately impeded classic “two hits” of NASH physiopathology. Red genes indicate increased expression; green genes indicate decreased expression. Normal arrows indicate activation. Arrows with a vertical line at the end indicate suppression.

LGZG is a classic TCM formula that has been effectively used to treat obesity and hyperlipidemia. Recently, it has exhibited potent effects on HFD-induced NAFLD [14]. In this study, we examined the comprehensive effects of LGZG on the hepatic global gene expression profile in NAFLD progression. Many of the changes in expression profile reflected decreased hepatic cholesterol, oxidative stress, and inflammation. Subsequently, the elevated gene expression in PPAR and Hh signaling pathways of NAFLD rats provided an assurance of RNA-seq dataset quality. Two potential mechanisms under the efficacy of LGZG for NAFLD were identified: (i) alleviated oxidative stress and (ii) promoted cholesterol secretion to reduce hepatic cholesterol accumulation (Figure 9). These observations indicated a “two-hits-targeting” [2, 21] mechanism for LGZG in the treatment of NAFLD.

### 4.1. Characteristics of HFD-Induced NAFLD

NAFLD physiopathology-associated pathways were significantly enriched with NAFLD-regulated genes. Among these pathways, activation of PPAR and Hh signaling pathways is of special interest because their deregulation contributes to liver damage and metabolic syndrome [22–27]. Hh signaling is significantly upregulated in NASH, compared with the normal healthy liver [28]. Recent study showed that activation of *SMO* could induce Hh-responsive hepatocytes in NAFLD [28], which lends strong support to our results (Figure 4(b)). Notably, multiple studies showed that in different rodent models of diet-induced NASH, pharmacological inhibition of *SMO* (vismodebig or LDE225) can deactivate Hh signaling pathway and consistently improve liver inflammation and fibrosis [25, 28–30]. Despite no significant difference was observed between NAFLD and LGZG treatment groups for *SMO*, a trend of decreased expression was shown in LGZG-treated NAFLD rats. In addition, lipid metabolism-related genes were markedly altered in NAFLD rats, which contributed to hepatic TG accumulation. Genes and pathways associated with NAFLD pathology were the manifestation of the RNA-seq dataset quality.

### 4.2. Alleviation of Oxidative Stress

Significant expression changes of four LGZG-regulated genes (*OSGIN1*, *AHR, IRF2BP2*, and *RASGEF1B*) exhibited alleviation of oxidative stress upon LGZG treatment, compared with the NAFLD group. As an antioxidant, *OSGIN1* is a cell growth inhibitor to resist oxidative stress [31]. Liu et al. reported that the mRNA level of *OSGIN1* was reduced in HCC specimens, and in HCC pateints, the inhibition of *OSGIN1* was related to shorter overall and disease-free survival times [32], suggesting that the significant upregulation of *OSGIN1* is critical for antioxidant response. *AHR* is identified to induce cellular oxidative stress and increase lipid peroxidation in NAFLD [33–36], and activation of *AHR* has pleotropic effects on steatosis of NAFLD [37, 38].

*RASGEF1B* and *IRF2BP2* were involved in inflammatory responses and were dramatically inhibited by the LGZG treatment. *RASGEF1B* was identified as a Ras-associated guanine nucleotide exchange factor and upregulated in macrophages stimulated with bacterial lipopolysaccharides (LPS) [39]. LPS is currently considered one of the major “hits” in NAFLD pathogenesis and progression [40]. Our current study showed significant upregulation of *RASGEF1B* in NAFLD model rats and reverse inhibition of *RASGEF1B* by LGZG treatment. Increased expression of *RASGEF1B* may be a strong defense response to LPS in NAFLD progression, and its restoration indicated the alleviation of inflammatory responses. *IRF2BP2* acts as a negative regulator of the nuclear factor of activated T cell (NFAT) transcription factor [41]. Restoration of *IRF2BP2* by LGZG treatment also suggested the alleviation of inflammatory responses in NAFLD. Continuous oxidative stress may lead to chronic inflammation. Thus, the alleviation of inflammatory responses also indicates the reduced oxidative stress upon LGZG treatment.

### 4.3. Activation of Cholesterol Secretion

Liver lipid accumulation is the first “hit” in the pathogenesis of NAFLD, and its removal is a desired intervention for NAFLD. Cholesterol is one type of important lipid in the liver, which has been shown as an emerging factor involved in the development of many metabolic diseases [8]. Free cholesterol stores in the liver in the formation of cholesterol esters. In the cholesterol secretion pathway, the upregulation of neutral cholesteryl ester hydrolase 1 (*NCEH1*) can accelerate the transformation of cholesterol esters into free cholesterol. *ABCG5* and *ABCG8* are two half-transporters that dimerize to create a cholesterol transporter, and their activation promotes the excretion of hepatic cholesterol [42]. In addition, *NCEH1*, *ABCG5*, and *ABCG8* are reported to be drug targets of pioglitazone [43] and ezetimibe [42] in the therapy of human gallbladder cholesterolosis and HFD-induced fatty liver.

## 5. Conclusion

Based on transcriptome analysis and experimental validation, our study examined the comprehensive effects of LGZG on hepatic global gene expression profile in HFD-induced NAFLD rats. Compared with normal rats, our data revealed significant upregulation of PPAR and Hh signaling pathways in NAFLD rats, which are known to be involved in NAFLD pathology and thus provide an assurance of the quality of our RNA-seq dataset. Of particular interest, NAFLD rats with LGZG treatment exhibited elevated expression of antioxidant and suppressed expression of prooxidant and proinflammatory genes in oxidative stress. Additionally, genes required for cholesterol secretion were increased by the LGZG treatment. These findings supported a “two-hits-targeting” mechanism for LGZG in the treatment of NAFLD: alleviating oxidative stress and activating cholesterol secretion.

## Figures and Tables

**Table 1:** Important LGZG-regulated genes.

Gene	*Q* value^∗^	FC^#^	*Q* value^$^	FC^+^	Associated function
*OSGIN1*	1.44e-03	0.43	5.10e-03	2.31	Oxidoreductase activity
*IRF2BP2*	6.62e-02	1.43	5.10e-03	0.52	Interferon regulatory factor 2-binding protein 1 & 2
*AHR*	2.20e-02	1.37	5.10e-03	0.67	Blood vessel development
*RASGEF1B*	1.44e-03	1.63	5.10e-03	0.46	Ras guanyl-nucleotide exchange factor activity

^∗^ *Q* value of comparison between the NAFLD model and normal groups, calculated from cuffdiff. ^#^Fold change of comparison between the NAFLD model and normal groups, calculated from cuffdiff. ^$^*Q* value of comparison between the LGZG-treated NAFLD and NAFLD model groups, calculated from cuffdiff. ^+^Fold change of comparison between the LGZG-treated NAFLD and NAFLD model groups, calculated from cuffdiff.

## Abbreviations

NAFLD: Nonalcoholic fatty liver disease
NASH: Nonalcoholic steatohepatitis
LGZG: Lingguizhugan
EBP/BW: Epididymal fat pad/body weight ratio
SPF: Specific pathogen free
HFD: High-fat diet
HPLC: High-performance liquid chromatography
H&E: Hematoxylin and eosin
ALT: Alanine aminotransferase
AST: Aspartate aminotransferase
TG: Triglyceride
TC: Total cholesterol
VLDL: Very-low-density lipoprotein
RT-PCR: Real-time PCR
DEG: Differentially expressed genes
SD: Standard deviation
ANOVA: One-way analysis of variance
CV: Central vein
PCSK9: Proprotein convertase subtilisin/kexin type 9
SQLE: Squalene monooxygenase
MGLL: Acylglycerol lipase
PLA2G7: Platelet-activating factor acetylhydrolase
IP6K1: Inositol hexakisphosphate 5-kinase
CXCL13: C-X-C motif chemokine 13
UBD: Ubiquitin D
SBSPON: Somatomedin B and thrombospondin type 1 domain containing
PPAR: Peroxisome proliferator-activated receptor
PPARG: Peroxisome proliferator-activated receptor gamma
SCD: Stearoyl-CoA desaturase
SCD2: Stearoyl-Coenzyme A desaturase 2
CYP7A1: Cholesterol 7alpha-monooxygenase
DBI: Diazepam-binding inhibitor (GABA receptor modulator, acyl-CoA-binding protein)
FABP1: Fatty acid binding protein 1, liver
CYP4A1: Alkane 1-monooxygenase
SMO: Smoothened
Hh: Hedgehog
ADRBK2: Beta-adrenergic receptor kinase
CCND2: Cyclin D2
INSIG1: Insulin-induced gene 1
LPIN1: Phosphatidate phosphatase LPIN
ABCG8: ATP-binding cassette, subfamily G (WHITE), member 8 (sterolin 2)
ABCG5: ATP-binding cassette, subfamily G (WHITE), member 5 (sterolin 1)
NCEH1: Neutral cholesterol ester hydrolase 1
OSGIN1: Oxidative stress-induced growth inhibitor 1
AHR: Aryl hydrocarbon receptor
IRF2BP2: Interferon regulatory factor 2 binding protein 2
RASGEF1B: RasGEF domain family, member 1B
HCC: Hepatocellular carcinoma.

## Supplementary Materials

Supplementary Materials Figure S1. Quality control of Lingguizhugan Decoction under High Performance Liquid Chromatography (HPLC). A. Reference substance; B. Lingguizhugan Decoction 1. glycyrrhizic acid. Standardization of extract by HPLC glycyrrhizic acid was purchased from Shanghai R&D Centre for standardization of Chinese Medicines (Shanghai, China), and the purity was higher than 98.0%;. HPLC-grade reagents were purchased from Burdick & Jackson. An Agilent 1100 HPLC system consisting of a G1354A pump, a G1313A auto-sampler, and a UV/VIS Photodiode Array G1315B Detector was used for all analyses. Chromatographic separations were carried out on an Merck C18 hibar column (4.6 mm × 250mm, 5 *μ*m) with methanol:acetonitrile : water :acetid acid(as solvent ) (15:35:45:0.9,v/v) in the mobile phase at a flow rate of 0.8 mL/min at 25°C for 40 min. Twenty *μ*L (after a high speed centrifugation) of the sample was injected, and the signals were detected at 254 nm with UV detection. The HPLC fingerprint of Lingguizhugan decoction revealed the major peaks (glycyrrhizic acid) at 254 nm. The content of glycyrrhizic acid were 0.9548%. Figure S2. Hierarchical clustering of replicate samples based on correlation matrix in three groups: (A) Normal group; (B) NAFLD model group; (C) LGZG-treated group. Each column and row represents samples in each group. Each cell of the heatmap reflects the distance of two samples. Distance was measured with Euclidean metric. The legend of distance value is shown on the upper right. Figure S2A and figure S2B revealed that s12 and s24 were the outlier samples of normal group and NAFLD model group, respectively. Figure S2C showed no outlier samples, but the data in s37 was missed during assessment of serum ALT and AST. Thus, s37 in LGZG-treated group was also removed in the RNA-seq analysis. Table S1. Primer pairs for real-time PCR analysis. Table S2. Enriched KEGG pathways with NAFLD regulated genes. Table S3. Enriched KEGG pathways with LGZG regulated genes.
