# Supplementary material for: Lingguizhugan Decoction Protects against High-Fat-Diet-Induced Nonalcoholic Fatty Liver Disease by Alleviating Oxidative Stress and Activating Cholesterol Secretion
Source: Int J Genomics. 2017 Dec 31;2017:2790864. doi: 10.1155/2017/2790864 (PMC5804362; doi:10.1155/2017/2790864)
Supplement: Supplementary Materials — Figure S1. Quality control of Lingguizhugan Decoction under High Performance Liquid Chromatography (HPLC). A. Reference substance; B. Lingguizhugan Decoction 1. glycyrrhizic acid. Standardization of extract by HPLC glycyrrhizic acid was purchased from Shanghai R&D Centre for standardization of Chinese Medicines (Shanghai, China), and the purity was higher than 98.0%;. HPLC-grade reagents were purchased from Burdick & Jackson. An Agilent 1100 HPLC system consisting of a G1354A pump, a G1313A auto-sampler, and a UV/VIS Photodiode Array G1315B Detector was used for all analyses. Chromatographic separations were carried out on an Merck C18 hibar column (4.6 mm × 250mm, 5 μm) with methanol:acetonitrile : water :acetid acid(as solvent ) (15:35:45:0.9,v/v) in the mobile phase at a flow rate of 0.8 mL/min at 25°C for 40 min. Twenty μL (after a high speed centrifugation) of the sample was injected, and the signals were detected at 254 nm with UV detection. The HPLC fingerprint of Lingguizhugan decoction revealed the major peaks (glycyrrhizic acid) at 254 nm. The content of glycyrrhizic acid were 0.9548%. Figure S2. Hierarchical clustering of replicate samples based on correlation matrix in three groups: (A) Normal group; (B) NAFLD model group; (C) LGZG-treated group. Each column and row represents samples in each group. Each cell of the heatmap reflects the distance of two samples. Distance was measured with Euclidean metric. The legend of distance value is shown on the upper right. Figure S2A and figure S2B revealed that s12 and s24 were the outlier samples of normal group and NAFLD model group, respectively. Figure S2C showed no outlier samples, but the data in s37 was missed during assessment of serum ALT and AST. Thus, s37 in LGZG-treated group was also removed in the RNA-seq analysis. Table S1. Primer pairs for real-time PCR analysis. Table S2. Enriched KEGG pathways with NAFLD regulated genes. Table S3. Enriched KEGG pathways with LGZG regulated genes. [file 2790864.f1.pdf]

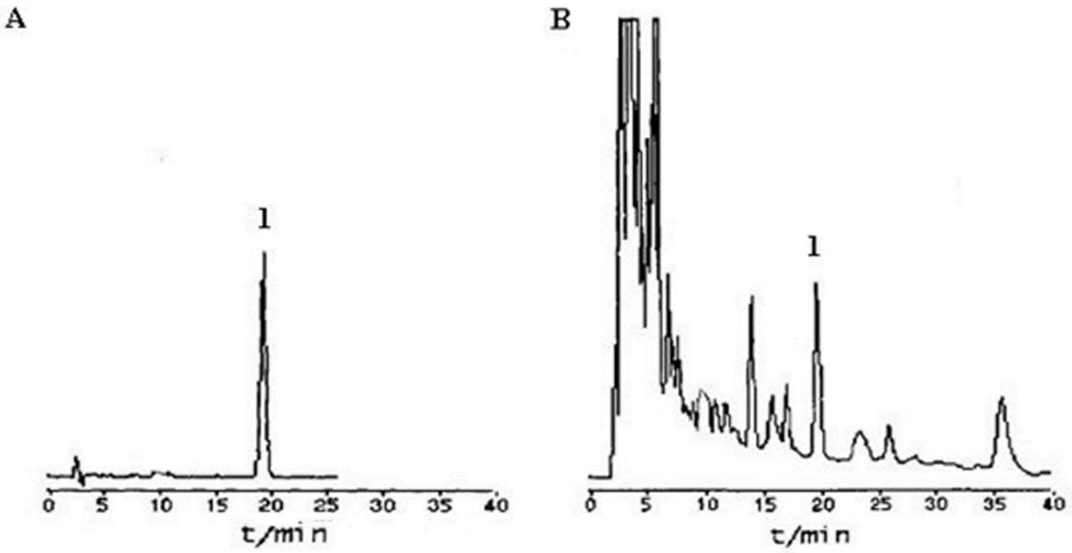

Figure S1. Quality control of *Lingguizhugan* Decoction under High Performance Liquid Chromatography (HPLC). A. Reference substance; B. *Lingguizhugan* Decoction 1. glycyrrhizic acid. Standardization of extract by HPLC glycyrrhizic acid was purchased from Shanghai R&D Centre for standardization of Chinese Medicines (Shanghai, China), and the purity was higher than 98.0%. HPLC-grade reagents were purchased from Burdick & Jackson. An Agilent 1100 HPLC system consisting of a G1354A pump, a G1313A auto-sampler, and a UV/VIS Photodiode Array G1315B Detector was used for all analyses. Chromatographic separations were carried out on an Merck C18 hibar column (4.6 mm×250mm， 5μm) with methanol:acetonitrile : water :acetid acid(as solvent) (15:35:45:0.9,v/v) in the mobile phase at a flow rate of 0.8 mL/min at 25℃ for 40 min. Twenty μL（after a high speed centrifugation）of the sample was injected, and the signals were detected at 254 nm with UV detection. The HPLC fingerprint of *Lingguizhugan* decoction revealed the major peaks (glycyrrhizic acid) at 254 nm. The content of glycyrrhizic acid were 0.9548%.

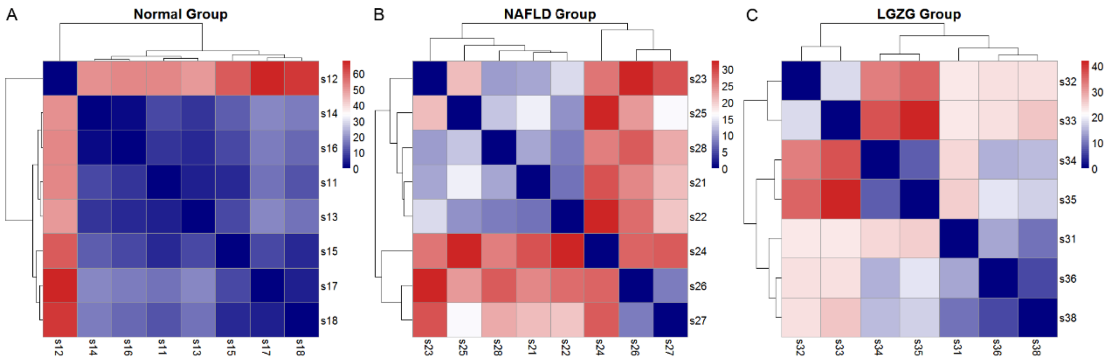

Figure S2. Hierarchical clustering of replicate samples based on correlation matrix in three groups: (A) Normal group; (B) NAFLD model group; (C) LGZG-treated group. Each column and row represents samples in each group. Each cell of the heatmap reflects the distance of two samples. Distance was measured with Euclidean metric. The legend of distance value is shown on the upper right. Figure S2A and figure S2B revealed that s12 and s24 were the outlier samples of normal group and NAFLD model group, respectively. Figure S2C showed no outlier samples, but the data in s37 was missed during assessment of serum ALT and AST. Thus, s37 in LGZG-treated group was also removed in the RNA-seq analysis.

Table S1.Primer pairs for real-time PCR analysis.

Gene	Forward primer	Reverse primer
OSGIN1	GCAGCAGATGATGCGTGAC	GGAGCCGATGAGGACGAG
IRF2BP2	CCCCATCACCCCTACATCC	GAACCTGGGTGGTATCTTTCG
AHR	GCCAATACGCACCAAAAGC	CCAACTGCCGCACTCACTC
RASGEF1B	CACCTCCCTTCTCAGCAATG	GCACCAAGTGTTGGATGAGC
β-ACTIN	CCCATCTATGAGGGTTACGC	TTTAATGTCACGCACGATTTC

Table S2. Enriched KEGG pathways with NAFLD regulated genes.

ID	KEGG Pathway	Gene Ratio*	Background Ratio[#]	P value[$]	Gene ID	Gene Count
rno00100	Steroid biosynthesis	8/355	20/8214	8.983E-07	Sqle/Msmo1/Cyp51/Dhcr7/Hsd17b7/Faxdc2/Dhcr24/Sc5d	8
rno05146	Amoebiasis	16/355	103/8214	7.631E-06	Adcy1/Serpinb9/Prkcb/Tlr4/Tlr2/Itgam/Itgb2/Lama4/Lama2/Plcb4/Serpinb6/Col4a1/Prkca/Actn1/Col4a2/Hspb1	16
rno04151	PI3K-Akt signalling pathway	32/355	336/8214	1.966E-05	Itga4/Osmr/Fgf1/Kitlg/Ddit4/Ccnd2/Col6a6/Cdkn1a/Tlr4/Tlr2/Reln/Itgb3/Creb3l2/Flt1/Thbs2/Syk/Lama4/Lama2/Kit/Tek/Col4a1/Prkca/Itga8/Csf1r/Col4a2/Kdr/Fgf21/LOC103692716/Efna1/Prlr/Gys2/Rps6kb2	32
rno01040	Biosynthesis of unsaturated fatty acids	8/355	29/8214	2.173E-05	Scd2/Scd1/Fads1/Elovl6/Acot5/Elovl2/Acot4/Acot7	8
rno01100	Metabolic pathways	87/355	1336/8214	2.9E-05	Pla2g7/Asns/Hdc/Cyp7a1/Phgdh/Psat1/Pycr1/Cyp2c22/Acsm5/Acsm3/Cyp3a2/Tusc3/Mthfd2/Tyms/Gatm/Cers6/Adh7/Pld1/Pla2g4a/Csgalnact1/Pkm/Aldh1b1/Plcb4/Papss1/Idh2/Gcnt1/Aspa/Pgap1/Rrm2b/Alg10/Akr1d1/Hk3/Pklr/Sqle/Mgll/Msmo1/Idi1/Cyp51/Cyp2c13/Mvd/Hmgcs1/Fdps/Dhcr7/Hsd17b7/Faxdc2/Cyp17a1/Acot5/Pmvk/Cyp2c12/Hmgcr/Dhcr24/Isyna1/Acot4/Acss2/Cyp2b1/Hexdc/Me3/Cyp4a1/Acacb/Amdhd1/Pdxp/Fasn/Cyp8b1/Fpgs/Cyp3a23/3a1/Agpat3/Impa2/Mvk/Dgkq/Upp2/Gale/Gls2/Nnmt/Itpka/Agxt/Pemt/Asl/Gys2/Galk1/Inpp5b/Ptgis/Sc5d/Ephx2/Got1/Pgp/Pgls/Me1	87
rno04621	NOD-like receptor signalling	20/355	168/8214	3.484E-05	Ccl2/Gbp5/Cxcl1/Tnfaip3/Gbp4/Gbp2/Ripk3/Tlr4/Nlrp1a/Ccl5/Plcb4/Xiap/Trpm2/Cybb/Nfkbia/Casp1/Irf3/LOC103692716/Mapk1	20

	pathway				1/Oas1a	
rno00 900	Terpenoid backbone biosynthesis	7/355	23/8214	3.571E-05	Idi1/Mvd/Hmgcs1/Fdps/Pmvk/Hmgcr/Mvk	7
rno00 140	Steroid hormone biosynthesis	13/355	83/8214	5.003E-05	Cyp7a1/Cyp2c22/Cyp7b1/Akr1c1/Cyp3a2/Akr1d1/Cyp2c13/Hsd17b7/Cyp17a1/Cyp2c12/Cyp2b1/Cyp3a23/3a1/Srd5a1	13
rno05 140	Leishmaniasis	12/355	74/8214	6.861E-05	RT1-Da/Itga4/RT1-DOb/RT1-Bb/RT1-Db1/Prkcb/Tlr4/Tlr2/Itgam/Itgb2/Nfkbia/Mapk11	12
rno05 152	Tuberculosis	20/355	185/8214	0.0001366	Clec7a/RT1-Da/Ciita/Cd74/Itgax/RT1-DOb/RT1-Bb/RT1-Db1/Tlr4/Tlr2/Itgam/Itgb2/Pla2r1/Ep300/Coro1a/Syk/Lsp1/Fcgr2b/Mrc1/Mapk11	20
rno05 164	Influenza A	19/355	171/8214	0.0001394	RT1-Da/Ciita/Ccl2/RT1-DOb/RT1-Bb/RT1-Db1/Prkcb/Tlr4/Ccl5/Ep300/Tnfsf10/Prkca/Nfkbia/Casp1/Fdps/Socs3/Irf3/Mapk11/Oas1a	19
rno04 510	Focal adhesion	21/355	204/8214	0.0001871	Itga4/Ccnd2/Col6a6/Prkcb/Reln/Cav2/Itgb3/Flt1/Thbs2/Parvg/Lama4/Lama2/Fyn/Vav1/Col4a1/Xiap/Prkca/Itga8/Actn1/Col4a2/Kdr	21
rno00 620	Pyruvate metabolism	8/355	39/8214	0.0002132	Me2/Pkm/Aldh1b1/Pklr/Acss2/Me3/Acacb/Me1	8
rno04 640	Hematopoietic cell lineage	13/355	97/8214	0.0002551	RT1-Da/Cd44/Itga4/Siglech/RT1-DOb/RT1-Bb/RT1-Db1/Cd7/Kitlg/Itgam/Itgb3/Kit/Csf1r	13
rno04 145	Phagosome	20/355	199/8214	0.0003647	Clec7a/RT1-Da/RT1-DOb/RT1-Bb/RT1-Db1/Tlr4/Tlr2/Msr1/Itgam/Itgb2/RT1-M3-1/Itgb3/Thbs2/Pla2r1/Coro1a/Dync1h1/Fcgr2b/Mrc1/Cybb/Tubb6	20
rno04 015	Rap1 signalling pathway	21/355	218/8214	0.0004647	Adcy1/Fgf1/Kitlg/Prkcb/Itgam/Itgb2/Itgb3/Flt1/Itgal/Gnai1/Plcb4/Kit/Tek/Rasgrp3/Magi3/Prkca/Csf1r/Kdr/Fgf21/Mapk11/Efna1	21

rno05323	Rheumatoid arthritis	12/355	92/8214	0.0005636	RT1-Da/Ccl2/RT1-DOb/RT1-Bb/RT1-Db1/Tlr4/Tlr2/Itgb2/Ccl5/Flt1/Itgal/Tek	12
rno04380	Osteoclast differentiation	15/355	133/8214	0.0005931	Lck/Blnk/Tnfrsf11b/Fosl2/Lilrb4/Itgb3/Syk/Fyn/Fcgr2b/Cybb/Csf1r/Pparg/Nfkbia/Socs3/Mapk11	15
rno04974	Protein digestion and absorption	12/355	93/8214	0.0006225	Cpb1/Slc1a5/Xpnpep2/Kcnn4/Col6a6/Slc8a1/Eln/Col4a1/Slc38a2/Col4a2/Kcnk5/Col27a1	12
rno01200	Carbon metabolism	14/355	123/8214	0.000818	Phgdh/Psat1/Me2/Pkm/Idh2/Hk3/Pklr/Acss2/Me3/Agxt/Got1/Pgp/Pgls/Me1	14
rno05145	Toxoplasmosis	14/355	123/8214	0.000818	RT1-Da/Ciita/RT1-DOb/RT1-Bb/RT1-Db1/Tlr4/Tlr2/Lama4/Lama2/Gnai1/Xiap/Nfkbia/Mapk11/Ldlr	14
rno04512	ECM-receptor interaction	11/355	85/8214	0.0010152	Cd44/Itga4/Col6a6/Reln/Itgb3/Thbs2/Lama4/Lama2/Col4a1/Itga8/Col4a2	11
rno04650	Natural killer cell mediated cytotoxicity	12/355	101/8214	0.0013068	Cd244/Lck/Prkcb/Itgb2/Prf1/Itgal/Syk/Cd48/Fyn/Vav1/Tnfsf10/Prkca	12
rno04670	Leukocyte transendothelial migration	13/355	118/8214	0.0016821	Itga4/Prkcb/Cdh5/Msn/Itgam/Itgb2/Itgal/Gnai1/Vav1/Prkca/Cybb/Actn1/Mapk11	13
rno03320	PPAR signalling pathway	10/355	82/8214	0.0026731	Cyp7a1/Scd2/Scd1/Pparg/Dbi/Cyp4a1/Plin5/Cyp8b1/Fabp1/Me1	10
rno05150	Staphylococcus aureus infection	8/355	57/8214	0.0029098	RT1-Da/RT1-DOb/RT1-Bb/RT1-Db1/Itgam/Itgb2/Itgal/Fcgr2b	8
rno00250	Alanine, aspartate and glutamate metabolism	6/355	35/8214	0.0035001	Asns/Aspa/Gls2/Agxt/Asl/Got1	6
rno04	Bile secretion	9/355	72/8214	0.0036435	Cyp7a1/Adcy1/Ephx1/Nr0b2/Abcg8/Hmgcr/Sult2a1/Ldlr/Abcg5	9

976						
rno04978	Mineral absorption	7/355	47/8214	0.0037507	Heph/Mt2A/Slc8a1/Slc34a2/Cybrd1/Slc39a4/Slc26a6	7
rno04514	Cell adhesion molecules (CAMs)	16/355	176/8214	0.003867	RT1-Da/Itga4/Sell/RT1-DOb/RT1-Bb/RT1-Db1/Spn/Ptprm/Cdh5/Itgam/Itgb2/RT1-M3-1/Itgal/Ptprc/Itga8/Icoslg	16
rno00120	Primary bile acid biosynthesis	4/355	16/8214	0.0041285	Cyp7a1/Cyp7b1/Akr1d1/Cyp8b1	4
rno04724	Glutamatergic synapse	12/355	116/8214	0.004227	Slc1a2/Adcy1/Adrbk2/Prkcb/Pld1/Pla2g4a/Gnai1/Plcb4/Prkca/Slc38a2/Grik5/Gls2	12
rno04146	Peroxisome	10/355	88/8214	0.0044882	Far1/Idh2/Pmvk/Crat/Pex11a/Mvk/Agxt/Pex6/Abcd3/Ephx2	10
rno04666	Fc gamma R-mediated phagocytosis	10/355	90/8214	0.005272	Prkcb/Pld1/Pla2g4a/Syk/Ptprc/Vav1/Fcgr2b/Prkca/Arpc1a/Rps6kb2	10
rno00062	Fatty acid elongation	5/355	29/8214	0.0073902	Elovl6/Acot5/Elovl2/Acot4/Acot7	5
rno05416	Viral myocarditis	10/355	95/8214	0.0077033	RT1-Da/RT1-DOb/RT1-Bb/RT1-Db1/Itgb2/RT1-M3-1/Prf1/Itgal/Lama2/Fyn	10
rno04910	Insulin signalling pathway	13/355	141/8214	0.0078511	Ppp1r3c/Srebf1/Foxo1/Pde3b/Hk3/Pklr/Socs3/Socs2/Acacb/Fasn/Prkab1/Gys2/Rps6kb2	13
rno04931	Insulin resistance	11/355	111/8214	0.0083129	Ppp1r3c/Srebf1/Prkcb/Foxo1/Creb3l2/Nfkbia/Socs3/Acacb/Prkab1/Gys2/Rps6kb2	11
rno00591	Linoleic acid metabolism	6/355	42/8214	0.0087809	Cyp2c22/Cyp3a2/Pla2g4a/Cyp2c13/Cyp2c12/Cyp3a23/3a1	6
rno04	AMPK signalling	12/355	129/8214	0.0097579	Scd2/Srebf1/Scd1/Foxo1/Creb3l2/Pparg/Hmgcr/Acacb/Fasn/Prka	12

152	pathway				b1/Gys2/Rps6kb2	
rno00830	Retinol metabolism	9/355	85/8214	0.0108065	Cyp2c22/Cyp3a2/Adh7/Lrat/Cyp2c13/Cyp2c12/Cyp2b1/Cyp4a1/Cyp3a23/3a1	9
rno04916	Melanogenesis	10/355	100/8214	0.0109206	Adcy1/Wnt2/Kitlg/Prkcb/Creb3l2/Ep300/Gnai1/Plcb4/Kit/Prkca	10
rno04360	Axon guidance	15/355	180/8214	0.0110703	Robo1/Smo/Unc5b/Unc5d/Nrp1/Ntn1/Sema6a/Dpysl2/Sema3d/Gnai1/Fyn/Prkca/Rgs3/Efna1/Sema6c	15
rno05134	Legionellosis	7/355	58/8214	0.0119691	Cxcl1/Tlr4/Tlr2/Itgam/Itgb2/Nfkbia/Casp1	7
rno04014	Ras signalling pathway	18/355	235/8214	0.0130606	Fgf1/Rasgrp1/Kitlg/Prkcb/Rasa3/Ets1/Pld1/Pla2g4a/Flt1/Rgl1/Kit/Tek/Rasgrp3/Prkca/Csf1r/Kdr/Fgf21/Efna1	18
rno04933	AGE-RAGE signalling pathway in diabetic complications	10/355	104/8214	0.0141574	Ccl2/Prkcb/Foxo1/Plcb4/Col4a1/Prkca/Cybb/Thbd/Col4a2/Mapk11	10
rno04672	Intestinal immune network for IgA production	6/355	49/8214	0.018201	RT1-Da/Itga4/RT1-DOb/RT1-Bb/RT1-Db1/Icoslg	6
rno04066	HIF-1 signalling pathway	10/355	109/8214	0.0191537	Cdkn1a/Prkcb/Tlr4/Flt1/Ep300/Tek/Prkca/Cybb/Hk3/Rps6kb2	10
rno05204	Chemical carcinogenesis	9/355	94/8214	0.019939	Cyp2c22/Ephx1/Cyp3a2/Adh7/Cyp2c13/Cyp2c12/Sult2a1/Cyp2b1/Cyp3a23/3a1	9
rno04668	TNF signalling pathway	10/355	110/8214	0.0202911	Ccl2/Cxcl1/Tnfaip3/Ripk3/Ccl5/Creb3l2/Ifi47/Nfkbia/Socs3/Mapk11	10
rno04	Cytokine-cytokine	17/355	229/8214	0.0205952	Cxcl13/Ccl2/Osmr/Csf2rb/Kitlg/Lifr/Tnfrsf11b/Cxcl9/Il13ra1/Tnf	17

060	receptor interaction				rsf21/Ccl5/Flt1/Kit/Tnfsf10/Csf1r/Kdr/Prlr	
rno04 062	Chemokine signalling pathway	14/355	177/8214	0.0209402	Cxcl13/Fgr/Ccl2/Adcy1/Cxcl1/Adrbk2/Prex1/Cxcl9/Prkcb/Ccl5/Gnai1/Plcb4/Vav1/Nfkbia	14
rno00 340	Histidine metabolism	4/355	25/8214	0.0211366	Hdc/Aldh1b1/Aspa/Amdhd1	4
rno04 940	Type I diabetes mellitus	8/355	80/8214	0.0217167	RT1-Da/RT1-DOb/RT1-Bb/RT1-Db1/RT1-M3-1/Prf1/Ica1/Ptprn	8
rno04 064	NF-kappa B signalling pathway	9/355	97/8214	0.0239562	Bcl2a1/Tnfaip3/Lck/Blnk/Prkcb/Tlr4/Syk/Xiap/Nfkbia	9
rno05 200	Pathways in cancer	26/355	400/8214	0.0239727	Adcy1/Smo/Fgf1/Wnt2/Rasgrp1/Kitlg/Cdkn1a/Prkcb/Foxo1/Ep300/Lama4/Lama2/Gnai1/Plcb4/Kit/Col4a1/Rasgrp3/Xiap/Prkca/Csf1r/Pparg/Nfkbia/Ar/Col4a2/Fgf21/LOC103692716	26
rno00 590	Arachidonic acid metabolism	8/355	83/8214	0.0264587	Cyp2c22/Pla2g4a/Cyp2c13/Cyp2c12/Cyp2b1/Cyp4a1/Ptgis/Ephx2	8
rno04 950	Maturity onset diabetes of the young	4/355	27/8214	0.0274148	Pklr/Onecut1/Foxa2/Hhex	4
rno01 212	Fatty acid metabolism	6/355	54/8214	0.0281176	Scd2/Scd1/Fads1/Elovl6/Elovl2/Fasn	6
rno04 750	Inflammatory mediator regulation of TRP channels	10/355	116/8214	0.0281731	Cyp2c22/Adcy1/Prkcb/Pla2g4a/Plcb4/Prkca/Cyp2c13/Cyp2c12/Cyp4a1/Mapk11	10

rno01230	Biosynthesis of amino acids	8/355	85/8214	0.0299938	Phgdh/Psat1/Pycr1/Pkm/Idh2/Pklr/Asl/Got1	8
rno00650	Butanoate metabolism	4/355	28/8214	0.0309279	Acsm5/Acsm3/Aacs/Hmgcs1	4
rno05166	HTLV-I infection	20/355	296/8214	0.031373	RT1-Da/RT1-DOb/RT1-Bb/RT1-Db1/Adcy1/Wnt2/Lck/Ccnd2/Cdkn1a/Nrp1/Ets1/Tp53inp1/Itgb2/RT1-M3-1/Itgal/Ep300/Xiap/Nfkbia/Fdps/Kat2a	20
rno04922	Glucagon signalling pathway	9/355	103/8214	0.0336619	Foxo1/Creb3l2/Ep300/Pkm/Plcb4/Pde3b/Acacb/Prkab1/Gys2	9
rno04918	Thyroid hormone synthesis	7/355	72/8214	0.0352378	Adcy1/Serpina7/Prkcb/Creb3l2/Plcb4/Prkca/Ttr	7
rno04540	Gap junction	8/355	88/8214	0.0358849	Adcy1/Prkcb/Gnai1/Plcb4/Prkca/Tubb6/Tjp1/Map2k5	8
rno05162	Measles	11/355	138/8214	0.0363064	Tnfaip3/Ccnd2/Tlr4/Tlr2/Msn/Fyn/Fcgr2b/Tnfsf10/Nfkbia/Irf3/Oas1a	11
rno04340	Hedgehog signalling pathway	5/355	43/8214	0.0367169	Smo/Adrbk2/Ccnd2/Ihh/Csnk1g2	5
rno05205	Proteoglycans in cancer	15/355	209/8214	0.0369739	Cd44/Smo/Wnt2/Cdkn1a/Prkcb/Tlr4/Tlr2/Cav2/Msn/Itgb3/Prkca/Kdr/Ihh/Mapk11/Rps6kb2	15
rno04662	B cell receptor signalling pathway	7/355	73/8214	0.0375916	Blnk/Prkcb/Syk/Vav1/Rasgrp3/Fcgr2b/Nfkbia	7
rno05202	Transcriptional misregulation in	13/355	174/8214	0.0381057	Bcl2a1/Ccnd2/Cdkn1a/Mef2c/Foxo1/Itgam/Flt1/Erg/Fli1/Csf1r/Pparg/Dusp6/Hhex	13

	cancer					
rno05133	Pertussis	7/355	74/8214	0.0400467	Tlr4/Itgam/Itgb2/Gnai1/Casp1/Irf3/Mapk11	7
rno05142	Chagas disease (American trypanosomiasis)	9/355	108/8214	0.0435959	Ccl2/Adcy1/Tlr4/Tlr2/Ccl5/Gnai1/Plcb4/Nfkbia/Mapk11	9
rno04370	VEGF signalling pathway	6/355	60/8214	0.0440443	Prkcb/Pla2g4a/Prkca/Kdr/Mapk11/Hspb1	6
rno05144	Malaria	6/355	60/8214	0.0440443	Ccl2/Tlr4/Tlr2/Itgb2/Thbs2/Itgal	6
rno04917	Prolactin signalling pathway	7/355	76/8214	0.045265	Ccnd2/Cish/Socs3/Cyp17a1/Socs2/Mapk11/Prlr	7

*Ratio of NAFLD regulated gene numbers annotated in this KEGG pathway to all gene numbers annotated in this KEGG pathway.

#Ratio of all gene numbers annotated in this KEGG pathway to all gene numbers annoated in all KEGG pathways.

$P value was calculated from hypergeometric test.

Table S3. Enriched KEGG pathways with LGZG regulated genes.

ID	KEGG Pathway	Gene Ratio*	Background Ratio#	P value$	Gene ID	Gene Count
rno04917	Prolactin signaling pathway	5/57	76/8214	0.0001708	Cish/Socs2/Gck/Pik3r1/Prlr	5
rno04910	Insulin signaling pathway	6/57	141/8214	0.0004064	Socs2/Ppp1r3c/Gck/Pik3r1/Fasn/Foxo1	6
rno04950	Maturity onset diabetes of the young	3/57	27/8214	0.0008235	Onecut1/Foxa2/Gck	3
rno04976	Bile secretion	4/57	72/8214	0.0015099	Abcg8/Abcg5/Nceh1/Nr0b2	4
rno04630	Jak-STAT signaling pathway	5/57	151/8214	0.0038136	Cish/Socs2/Pik3r1/Cdkn1a/Prlr	5
rno04930	Type II diabetes mellitus	3/57	52/8214	0.0055043	Socs2/Gck/Pik3r1	3
rno01212	Fatty acid metabolism	3/57	54/8214	0.0061177	Fasn/Scd1/Elovl5	3
rno04213	Longevity regulating pathway - multiple species	3/57	65/8214	0.0102107	Foxa2/Pik3r1/Foxo1	3
rno04152	AMPK signaling pathway	5/57	129/8214	0.0120739	Pik3r1/Fasn/Foxo1/Scd1/Foxo3	4
rno04115	p53 signaling pathway	3/57	71/8214	0.0129762	Rprm/Cdkn1a/Gadd45g	3
rno05218	Melanoma	3/57	71/8214	0.0129762	Fgf21/Pik3r1/Cdkn1a	3
rno040	FoxO signaling pathway	5/57	136/8214	0.01442	Pik3r1/Foxo1/Cdkn1a/Gadd45g/Fox	4

68				91	o3/Bcl2l11	
rno010<br>40	Biosynthesis of unsaturated fatty acids	2/57	29/8214	0.01703<br>66	Scd1/Elovl5	2
rno045<br>50	Signaling pathways regulating pluripotency of stem cells	4/57	143/8214	0.01705<br>83	Onecut1/Pik3r1/Fzd1/Inhba	4
rno047<br>10	Circadian rhythm	2/57	30/8214	0.01817<br>31	Cry1/Cry2	2
rno052<br>24	Breast cancer	4/57	148/8214	0.01910<br>92	Fgf21/Pik3r1/Cdkn1a/Fzd1	4
rno005<br>00	Starch and sucrose metabolism	2/57	32/8214	0.02053<br>94	Pgm2l1/Gck	2
rno052<br>15	Prostate cancer	3/57	89/8214	0.02362<br>82	Pik3r1/Foxo1/Cdkn1a	3
rno041<br>50	mTOR signaling pathway	4/57	160/8214	0.02463<br>79	Lpin1/Pik3r1/Fzd1/Ddit4	4
rno049<br>75	Fat digestion and absorption	2/57	40/8214	0.03118<br>2	Abcg8/Abcg5	2
rno052<br>02	Transcriptional misregulation in cancer	4/57	174/8214	0.03220<br>36	Foxo1/Cdkn1a/Slc45a3/Aff1	4
rno040<br>10	MAPK signaling pathway	5/57	259/8214	0.03314<br>92	Dusp10/Fgf21/Il1b/Gadd45g/Dusp1	5
rno005<br>91	Linoleic acid metabolism	2/57	42/8214	0.03411<br>91	Cyp3a23/3a1/Cyp2j4	2
rno049<br>33	AGE-RAGE signaling pathway in diabetic complications	3/57	104/8214	0.03524<br>27	Il1b/Pik3r1/Foxo1	3
rno043	Axon guidance	4/57	180/8214	0.03582	Rgs3/Ablim3/Pik3r1/Dpysl2	4

60				2		
rno04931	Insulin resistance	3/57	111/8214	0.0415065	Ppp1r3c/Pik3r1/Foxo1	3
rno02010	ABC transporters	2/57	47/8214	0.0419084	Abcg8/Abcg5	2
rno04750	Inflammatory mediator regulation of TRP channels	3/57	116/8214	0.0463028	Il1b/Cyp2j4/Pik3r1	3
rno00400	Phenylalanine, tyrosine and tryptophan biosynthesis	1/57	7/8214	0.047593	Tat	1

*Ratio of LGZG regulated gene numbers annotated in this KEGG pathway to all gene numbers annotated in this KEGG pathway.

#Ratio of all gene numbers annotated in this KEGG pathway to all gene numbers annoated in all KEGG pathways.

$P value was calculated from hypergeometric test.
